# Assessment of artificial intelligence model and manual morphokinetic annotation system as embryo grading methods for successful live birth prediction: a retrospective monocentric study

**DOI:** 10.1186/s12958-024-01198-7

**Published:** 2024-03-05

**Authors:** Myrto-Sotiria Papamentzelopoulou, Ilectra-Niki Prifti, Despoina Mavrogianni, Thomais Tseva, Ntilay Soyhan, Aikaterini Athanasiou, Antonia Athanasiou, Adamantios Athanasiou, Paraskevi Vogiatzi, George Konomos, Dimitrios Loutradis, Maria Sakellariou

**Affiliations:** 1 Molecular Biology Unit, Division of Human Reproduction, 1st Department of Obstetrics and Gynecology, National and Kapodistrian University of Athens, 80, Vasilissis Sofias Ave., Athens, 11528 Greece; 2 IVF Athens Reproduction Center V. Athanassiou, Maroussi, Greece; 3 HUG (Hôpitaux universitaires de Genève), Rue Gabrielle-Perret-Gentil 4, Genève 14, Genève, 1211 Switzerland; 4 RHNe (Réseau hospitalier neuchâtelois), Chasseral 20, La Chaux-de-Fonds, 2303 Switzerland; 5 Department of Gynecology Oncology, Agios Savvas, General Anti-Cancer Hospital, Athens, Greece; 6 Andromed Health & Reproduction, Fertility Diagnostics Laboratory, Maroussi, Greece; 7 LE MONDE College, Athens, Greece; 8 Institute of Fertility, Athens, Greece

**Keywords:** Artificial intelligence, Time-lapse monitoring, IVF outcome, KIDScore, iDAScore, Embryo selection

## Abstract

**Purpose:**

The introduction of the time-lapse monitoring system (TMS) and the development of predictive algorithms could contribute to the optimal embryos selection for transfer. Therefore, the present study aims at investigating the efficiency of KIDScore and iDAScore systems for blastocyst stage embryos in predicting live birth events.

**Methods:**

The present retrospective study was conducted in a private IVF Unit setting throughout a 10-month period from October 2021 to July 2022, and included the analysis of 429 embryos deriving from 91 IVF/ICSI cycles conducted due to infertility of various etiologies. Embryos incubated at the Embryoscope^+^ timelapse incubator were analyzed through the established scoring systems: KIDScore and iDAScore®. The main outcome measure was the comparison of the two scoring systems in terms of live birth prediction. Embryos with the higher scores at day 5 (KID5 score/iDA5 score) were transferred or cryopreserved for later use.

**Results:**

Embryos with high KID5 and iDA5 scores positively correlated with the probability of successful live birth, with KID5 score yielding a higher efficiency in predicting a successful reproductive outcome compared to a proportionally high iDA5 score. KID5 demonstrated conservative performance in successfully predicting live birth compared to iDA5 score, indicating that an efficient prediction can be either provided by a relatively lower KID5 score or a relatively higher iDA5 score.

**Conclusion:**

The developed artificial intelligence tools should be implemented in clinical practice in conjunction with the conventional morphological assessment for the conduction of optimized embryo transfer in terms of a successful live birth.

## Introduction

In vitro fertilization (IVF) outcome is mainly associated with oocyte and embryo quality, since these factors can successfully predict reproductive outcomes [1]. Optimized selection of the most competent embryos for transfer to the endometrium has been one of the major challenges in assisted reproduction. Despite the notable advances in tailoring ovarian stimulation or the significant improvements in culture conditions and the application of implantation genetic analysis, only one-third of all IVF cycles result in pregnancy [2]. The introduction of time-lapse monitoring systems (TLSs) has enabled a consistent monitoring of embryo kinetics by recording the exact time-points of embryo divisions and the morphological changes [3]. The analysis of the respective data, in turn, enabled the construction of predictive algorithms for embryo selection in more terms than microscopical observation [4]. The goal of these models were to predict embryo quality [5, 6], embryonic genetic integrity [7], impantation [8] and embryo progression to live birth [9]. To date, several studies have demonstrated an improvement in clinical outcomes when time-lapse is applied for embryo morphokinetic selection, as compared to conventional incubation and embryological observation [10, 11]. In contrast, other studies propose an interlaboratory validation of models before use [12]. Currently, these methods are applied in conjunction with conventional approaches, mainly as a surrogate tool for categorizing or selecting embryos for transfer or cryopreservation.

On this basis, computerized algorithms have been incorporated to predictive software which accompanies newest versions of TLS incubators, thus assisting laboratory routine in embryo evaluation by providing an automated scoring of the embryos. Additionally, the continuous recording of data of embryonic development inside the incubator ensures a stable culture environment by limiting environmental changes and disruptions [5, 13].

The first TLS (Primo Vision^™^, Vitrolife, Göteborg, Sweden) was introduced at the ESHRE meeting in 2008. Since then many different TLSs incubators have been developed and are available for use in IVF units, including the FDA-approved Embryoscope^+^ (Vitrolife, Göteborg, Sweden). EmbryoScope^+^ integrates embryological data from multiple centers worldwide, on multiple time points to improve statistical significance and provides automatic detection image patterns to identify the top quality embryos within a patient’s cohort [10].

EmbryoViewer software (Vitrolife, Göteborg, Sweden) that accompanies Embryoscope^+^ offers two different scoring systems. KIDScore (Known Implantation Data), which is a manual morphokinetic annotation scoring system for either day 3 and/or day 5 embryos, and iDA Score, a fully automated blastocyst selection software through which the operators address intrinsic inter and intra variability. KIDScore decision support tool has been developed by analyzing the world’s largest database of embryo development with known clinical outcome and it combines manual annotation and AI. The models are developed by analyzing how embryo morphokinetics, cleavage patterns and morphology correlate with implantation outcome after embryo transfer. For each embryo the model calculates a continuous score from 1 to 9.9. The higher the score, the greater the statistical chance of implantation. Two different scoring systems are available (KIDScore D3/ KIDScore D5) depending on the stage of the embryo (Day3/ Day5). In our study we have focused on KIDScore D5 scoring system which reflects the statistical chance of implantation based on development information from the 5/6-day culture period. iDAScore algorithm was developed by Vitrolife’s AI team and trained on full time-lapse sequences of more than 180,000 embryos with known clinical fate and it is based on a 3D convolutional neural network [14].

The aim of the present study was to investigate the efficiency of KIDScore and iDAScore for blastocyst stage embryos in predicting live birth events, in order to directly evaluate the possible imbalances between operator’s subjectivity and artificial intelligence.

## Materials and methods

### Clinical setting, study design and criteria for participation in the study

The present retrospective study presents embryological and clinical data from an experienced private Assisted Reproduction Unit, “IVF Athens Reproduction Center” in Athens, Greece, collected throughout October 2021 to July 2022. The study was approved by the Research and Ethics Committee of the IVF Unit (EVD1003/2022) and was conducted in accordance with the ethical standards of the National Authority for Medically Assisted Reproduction and the 1964 Helsinki Declaration and its later amendments [15].

Data were collected from 91 subsequent IVF/ICSI cycles in a matching number of infertile patients that were conducted following to infertility diagnosis of various etiologies: female/male factor or combined, unexplained infertility and repeated implantation failure following IVF/ICSI. Patients received extensive consultation throughout the stages of treatment and consented to the treatment regime. Female partners had an average age of 34.42 ± 3.34 years (min = 23 years; max = 40 years), while 429 blastocyst stage embryos were analyzed.

Exclusion criteria included IVF/ICSI cycles conducted with embryo cultures in conventional incubators, cycles with embryo transfer and/or cryopreservation at earlier stages (day 2 or day 3), early embryo arrested development and those with incomplete data that failed to follow-up. Cycles with donor gametes (donor sperm and/or donor oocyte), surrogacy or embryo biopsy for preimplantation genetic analysis for aneuploidies (PGT-A), monogenic disorders (PGT-M) or structural rearrangements (PGT-SR), were also excluded. Moreover, female participants with any endometrial or endocrinological pathology and/or any medical history of endometriosis, hydrosalpinx, or autoimmune disorders were excluded. Male partners with genitourinary infection or other reproductive pathologies, with medical history of malignancies or previous chemotherapy and/or radiotherapy were also excluded. Cycles that received adjuvant treatments, or cycles with obstructive or non-obstructive azoospermia or with absolute terazoospermia (0% typical forms in the ejaculate) according to the applied WHO strict criteria [16, 17] were not included in the present study.

### Ovarian stimulation, oocyte retrieval and ICSI

For all the included cycles in the study, patients underwent an antagonist protocol for controlled ovarian stimulation initiated at day 2 to 4 of the menstrual cycle with recombinant FSH (Gonal F, MerkKGaA, Darmstadt, Germany; or Puregon (MSD, Kenilworth, NJ, USA), alone or in combination with urinary gonadotropins (hMG) (Menopur, Ferring, Saint-Prex Switzerland) and the use of antagonist Cetrotide 0.25 mg (Merck, MerkKGaA, Darmstadt, Germany) or Orgalutran 0.25 mg (Organon, Oss, Netherlands) when the leading follicle reached 14 mm and up to the day of final triggering. Ovarian response was monitored by transvaginal ultrasound with assessments of follicular growth, serum estradiol and progesterone levels every 1–3 days during stimulation. FSH and hMG dosages were adjusted accordingly to reach an optimal oocyte retrieval rate for each case.

When leading follicles reached at least 17 mm, ovulation induction was performed by administering 250 µg of choriogonadotropin alpha (Ovitrelle, Merck Serono Europe Limited, London, UK), while oocyte retrieval was performed 34–36 h after triggering under general anesthesia. Retrieved oocytes were reserved in a conventional incubator (Labotect, C200) in pre-equilibrated culture medium dishes (Universal IVF Medium, Origio a/s, Malov, Denmark) covered with mineral oil (OVOIL, Vitrolife, Sweden) at stable conditions of 5.0% O2, 6.6% CO_2_ and 37^ο^C [18] until cumulus denudation and insemination by ICSI. Sperm preparation and assessment was performed according to our previous publication [19] and conformed to the WHO procedures for gamete handling [17].

Ooocyte fertilization through ICSI was performed approximately 40 h after beta-hCG administration. Following sperm injection, oocytes were transferred into the microwells of the pre-equilibrated specialized embryo culture dish (EmbryoSlide^+ TM^, Vitrolife A/S, Viby, Denmark) that contained single-step culture media (Sage 1-Step, Origio a/s, Malov, Denmark) covered by mineral oil (OVOIL, Vitrolife, Sweden), throughout day 5 to early day 6 of embryo development.

### Embryo culture and time-lapse embryo assessment

All embryos were cultured in the FDA-approved Embryoscope^+^ incubator (Vitrolife, A/S, Viby, Denmark) installed with the EmbryoViewer software 7.8.2 (Vitrolife, A/S, Denmark). Collection and analysis of patient data was conducted anonymously by using specifically allocated reference codes and without any dominant of personal identification. Embryos with normal fertilization (appearance of two pronuclei) that progressed to blastocyst formation up to early day 6 were included in the analysis. Image sequences were acquired throughout the period of embryo culture via EmbryoViewer v.7.8.2 (Vitrolife, A/S, Denmark) according to the manufacturer’s settings at 10 min intervals in 11 focal planes. The accompanying software provided the implementation of time-lapse-based embryo analysis by the scoring systems of KIDScore and iDAScore® (Vitrolife, A/S, Viby, Denmark).

For KIDScore D5, all embryos were annotated by two trained and experienced embryologists in order to eliminate the intrinsic inter- and intra- reader variability, according to current guidelines [20–22]. For all embryos, the following information was recorded for the application of KIDScore D5: number of pronuclei (PN), timing of syngamy (tPNf), t2 (time from insemination to complete division to two cells), t3 (time from insemination to complete division to three cells), t5 (time from insemination to complete division to five cells), t8 (time from insemination to complete division to eight cells), tB (time from insemination to formation of blastocyst), ICM (Inner cell mass evaluation) and TE (Trophectoderm evaluation). Irregular morphokinetic events (such as reverse cleavage, multinucleation, abnormal pronuclei) were monitored, giving the advantage to deselect these embryos [23, 24].

iDAScore v1.2.0 software (Intelligent Data Analysis Score, Vitrolife, A/S, Denmark) provides a fully automated analysis of time-lapse sequences from the time of insemination (t0) until blastocyst stage development (108–148 h post-insemination). A higher score indicates a greater chance of achieving successful events of clinical pregnancy with positive fetal heartbeat. A score from 1 (lowest) to 9.9 (highest) is automatically generated for each embryo which is statistically correlated with its implantation potential. iDAScore provides a final grading for each embryo without being influenced by the evaluation of the operator [25], thus, eliminating the subjectivity of the conventional observatory approach. No patient data (e.g., age) or morphokinetic parameters are used as input to this model.

In freeze-all cycles or in cycles with surplus cryopreserved embryos, good quality blastocysts (GQBs) as defined by embryologists and according to Gardner’s criteria, were vitrified on day 5 if these were presented with a good quality inner cell mass (ICM) and trophectoderm (TE). Embryos not reaching adequate expansion or not meeting the above-mentioned criteria remained in culture an additional day and according to their developmental characteristics were vitrified on day 6. Blastocysts were cryopreserved by vitrification according to the protocols implementated in the IVF Unit routine practice, and according to manufacturer’s procedures for Vit Kit Freeze/Warm NX (FUJIFILM Irvine Scientific, INC, Santa Ana, CA, USA). Embryos with the highest scores were selected to be transferred first in fresh cycles or prioritized for future transfer after vitrification and warming. A maximum of three embryos per transfer was optioned if possible as allowed by the National Legislation Authority [26].

### Embryo transfer and clinical outcomes

All included cycles in the analysis had either fresh or frozen embryo replacement (FER) using vitrified/warmed embryos. For the fresh embryo transfers (ET), luteal support was provided by intramuscular progesterone injection (Prolutex, IBSA Farmaceutici Italia, Lodi, Italy), whereas, for frozen embryo replacement (FER) patients were prepared through a combination of oral capsules (Utrogestan, Faran Laboratories AVEE, Attica, Greece) and intramuscular injection (Prolutex, IBSA Farmaceutici Italia, Lodi, Italy). ET was performed under trans-abdominal ultrasound guidance for adequate embryo deposition with Wallace catheters (CooperSurgical, Malov, Denmark) either on day 5 of embryonic development in fresh ET cycles or after 6 days of progesterone administration in FER by ensuring a receptive endometrium. Serum human chorionic gonadotropin levels were measured 14 days after ET to confirm biochemical pregnancy. A clinical pregnancy was assured by ultrasonographic visualization with the presence of intrauterine gestational sac/s with confirmed fetal heart activity 6 weeks following ET. The main outcome measure was the live birth prediction, as it is considered the strongest endpoint in assisted reproduction.

### Data collection and analysis

Collection and analysis of patient data was conducted anonymously by using specifically allocated reference codes and without any dominant of personal identification. For all embryos at blastocyst stage, KIDScore D5 annotation and iDAScore evaluation have been recorded for statistical analysis and review in terms of reproductive outcome prediction. For the statistical analysis, descriptive statistics of the data provided an essential summary of the basic features of included population and its characteristics. Pearson correlation was subsequently carried out for the evaluation of the linear relationship between KID5 and iDA5 score. Simple logistic regression for KID5 score and iDA5 score in terms of live birth was performed. Paired t-test was applied in order to investigate the extent of KID5 and iDA5 scores difference in contrast to the probability of live birth. Multiple logistic regression was applied to explore KID5 and iDA5 scores and live birth probability, adjusted for age and number of blastocysts, followed. ROC (receiver operating characteristic) analysis was performed in order to evaluate KID5 and iDA5 score performance, with ROC curves at all possible classification thresholds designed for each score. The statistical analysis and graphical representations were carried out using SPSS version 20.0 (IBM SPSS Statistics). Outcomes were considered statistically significant when p-value was < 0.05.

## Results

### Study group descriptive statistics

The present study aimed at assessing the score predictions from the manual morphokinetic annotation system (KID Score) and the proposed AI model (iDA Score, 7:8.2) in grading blastocysts according to their developmental potency. Ninenty-one IVF/ICSI cycles were included with an average age of female partner of 34.42 ± 3.34 years (min = 23 years; max = 40 years). In total the transitional events of 429 blastocysts were recorded and analyzed in terms of the respective clinical outcome.

**Fig. 1:**
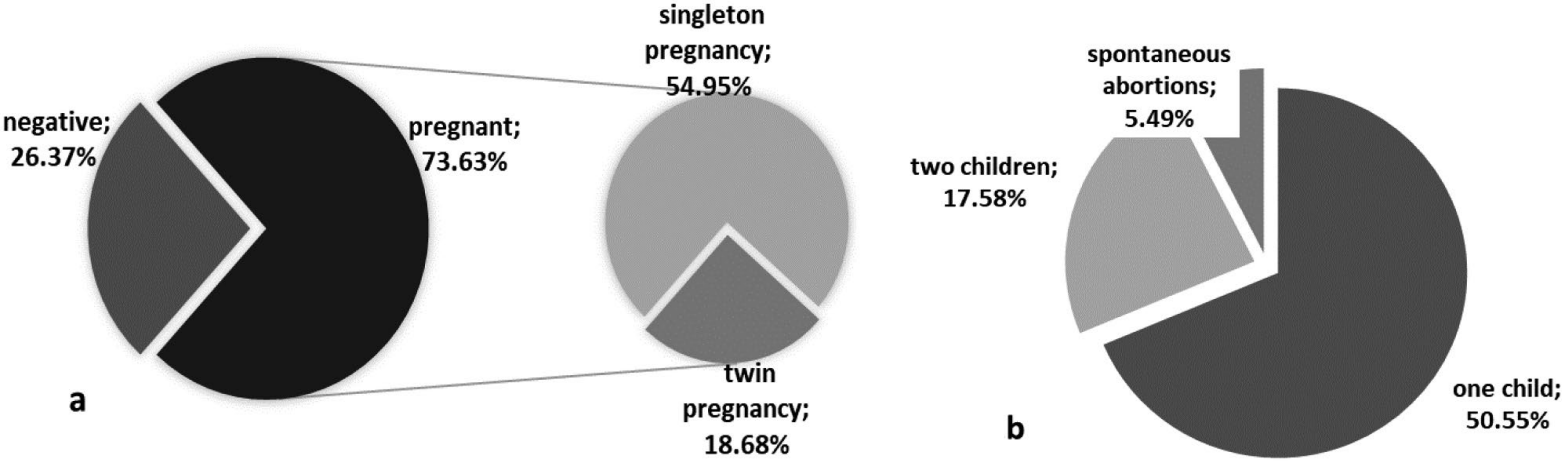
IVF outcome-related descriptive statistics for the 91 participants. (1**a**) pregnancy status; (1**b**) number of born children and delivery status

Of the participants, 31.9% (29/91) had fresh ET, 64.8% (59/91) frozen ET, and 3.3% (3/91) both fresh and frozen ET. Single embryo transfer (sET) was conducted in 20.88% (19/91), while in 76.92% (70/91) of the cases two embryos were transferred and 2.20% (2/91) had three embryos available for ET, since both had more than three unsuccessful previous IVF cycles. Regarding clinical outcomes, 73.63% (67/91) of the total cohort achieved a clinical pregnancy while 26.37% (24/91) of the participants had negative serum beta-hCG following ET (Fig. 1a). Singleton pregnancies comprised the 54.95% (50/91) of total pregnancy outcomes and in the remaining 18.68% (17/91) two fetal sacs and two distinct FHBs were present upon ultrasound examination (Fig. 1a).

In the subgroup analysis of cases women with sET, 42.10% (8/19) became pregnant, while in the group of two or three transferred embryos 58.33% (42/72) achieved a singleton pregnancy and 23.61% (17/72) had a twin pregnancy. Ultimately, 50.55% (46/91) gave birth to one child, 17.58% (16/91) gave birth to two children, while 5.49% (5/91; 4 singletons and 1 twin pregnancy) had spontaneous abortions or miscarriages due to abnormal chromosomal status of the fetus (Fig. 1b).

Regarding the scoring systems, the average KID Score at day 5 (KID5 score) was 7.31 ± 1.78 (min = 1.70; max = 9.60), while the average iDA5 Score at day 5 (iDA5 score) was 8.17 ± 1.36 (min = 3.50; max = 9.60).

### KID5 and iDA5 scores correlations

A statistically significantly robust, positive linear correlation between KID5 and iDA5 average scores was identified (p-values < 0.001), indicating that when KID5 score increases iDA5 score proportionally increases and that their predictions agree and correlate with each other. Such finding is also supported by partial correlations exhibiting similarly strong, positive associations between KID5 and iDA5 scores with live birth rate and successful pregnancy rate being control variables (p-values < 0.001).

### KID5 and iDA5 scores associations with successful birth probability

Simple logistic regression revealed statistically significant positive correlations between KID5 score and the probability of live birth (OR = 1.651, 95% CI [1.213–2.247]; p-value = 0.001, Table 1). A similar statistically significant correlation was observed between iDA5 score and the probability of live birth (OR = 1.619, 95% CI [1.111–2.359]; p-value = 0.012, Table 1). Accordingly, high KID5 or iDA5 scores are associated with increased probability of live birth.

**Table 1:** Simple logistic regression for KID5 and iDA5 scores and successful live birth probability

	coef	p-value	OR	95% CI for OR
KID5 score	0.501	0.001	1.651	[1.213–2.247]
constant	-3.043	0.008	0.048	
iDA5 score	0.482	0.012	1.619	[1.111–2.359]
constant	-3.346	0.033	0.035	

**Fig. 2:**
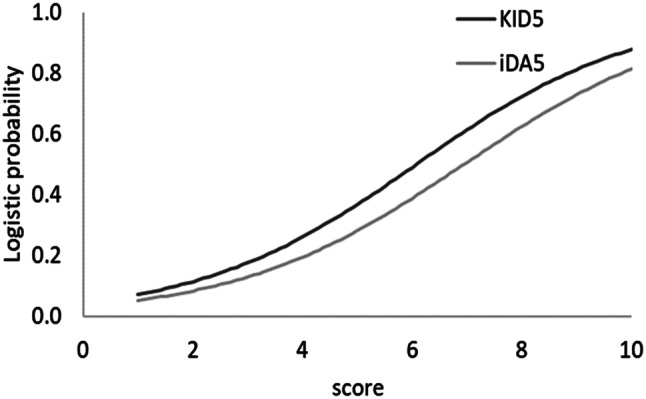
Logistic probability for successful live birth in relation to KID5 and iDA5 scores

Moreover, an increase in either KID5 or iDA5 score results in optimized probability of successful birth; however, KID5 score yields higher probability and predictive capacity of live birth compared to iDA5 at a given score, as demonstrated in Fig. 2. Following that observation, paired t-test was performed in order to investigate to what extent KID5 and iDA5 scores differ regarding the probability of live birth. As demonstrated, KID5 score yields a statistically significantly higher average probability for the prediction of live birth compared to iDA5 score (p-value < 0.001, Table 2). Therefore, it is more probable to successfully predict a live birth via KID5 score.

**Table 2:** Paired t-test for average logistic probability of successful live birth in relation to KID5 and iDA5 scores

	Average Logistic Probability	SD	p-value
KID5 score	0.45	0.27	< 0.001
iDA5 score	0.38	0.25	

Moreover, KID5 and iDA5 score effects were compared in terms of probability for successful birth outcome, adjusted for maternal age and number of blastocysts. As presented in Table 3, KID5 score exhibits a significant effect on the probability for birth outcome, regardless of maternal age and number of blastocysts (p-value = 0.010). Such finding suggests that embryo selection through high KID5 scores increases the probability of a successful clinical outcome. On the contrary, iDA5 score has no statistically significant effect on the probability for birth outcome and appears to depend on other variables, such as maternal age and number of blastocysts (Table 3, p-value = 0.062).

**Table 3:** Binary logistic regression for KID5 and iDA5 scores and successful live birth probability, adjusted for maternal age and number of blastocysts

Variables	coef	p-value	OR	95% CI for OR
KID5 score	0.423	0.010	1.051	[1.107–2.103]
Num of BCs	0.165	0.092	1.180	[0.974–1.430]
Maternal age	0.049	0.533	1.526	[0.900–1.226]
Constant	-4.902	0.111	0.007	
iDA5 score	0.368	0.062	1.446	[0.981–2.130]
Num of BCs	0.192	0.050	1.211	[1.000–1.468]
Maternal age	0.056	0.476	1.057	[0.907–1.231]
Constant	-5.179	0.118	0.006	

BCs: Blastocysts; Num.: Number

**Fig. 3:**
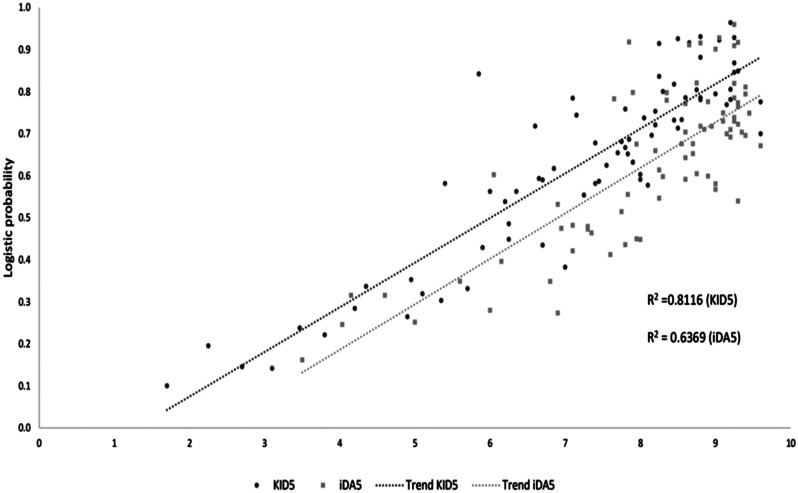
Logistic probability for successful birth outcome in relation to KID5 and iDA5 scores, adjusted for maternal age and number of blastocysts. (Bcs = Blastocysts)

KID5 score exhibits a positive correlation with the probability for live birth adjusted for maternal age and number of blastocysts is also illustrated in Fig. 3. The parallel trendlines for KID5 and iDA5 scores reveal that the difference of the two scores is independent of the maternal age and the number of blastocysts, indicating that KID5 and iDA5 scores are co-modified. Moreover, KID5 score seems to be underestimated compared to iDA5 score in a given birth probability, since KID5 yields more conservative scores. Accordingly, iDA5 score is presented as overestimated in a given birth probability adjusted for maternal age and number of blastocysts, e.g., a birth probability of 0.4 corresponds to an iDA5 score of 6, while KID5 score is calculated at 5.

### ROC analysis

**Fig. 4:**
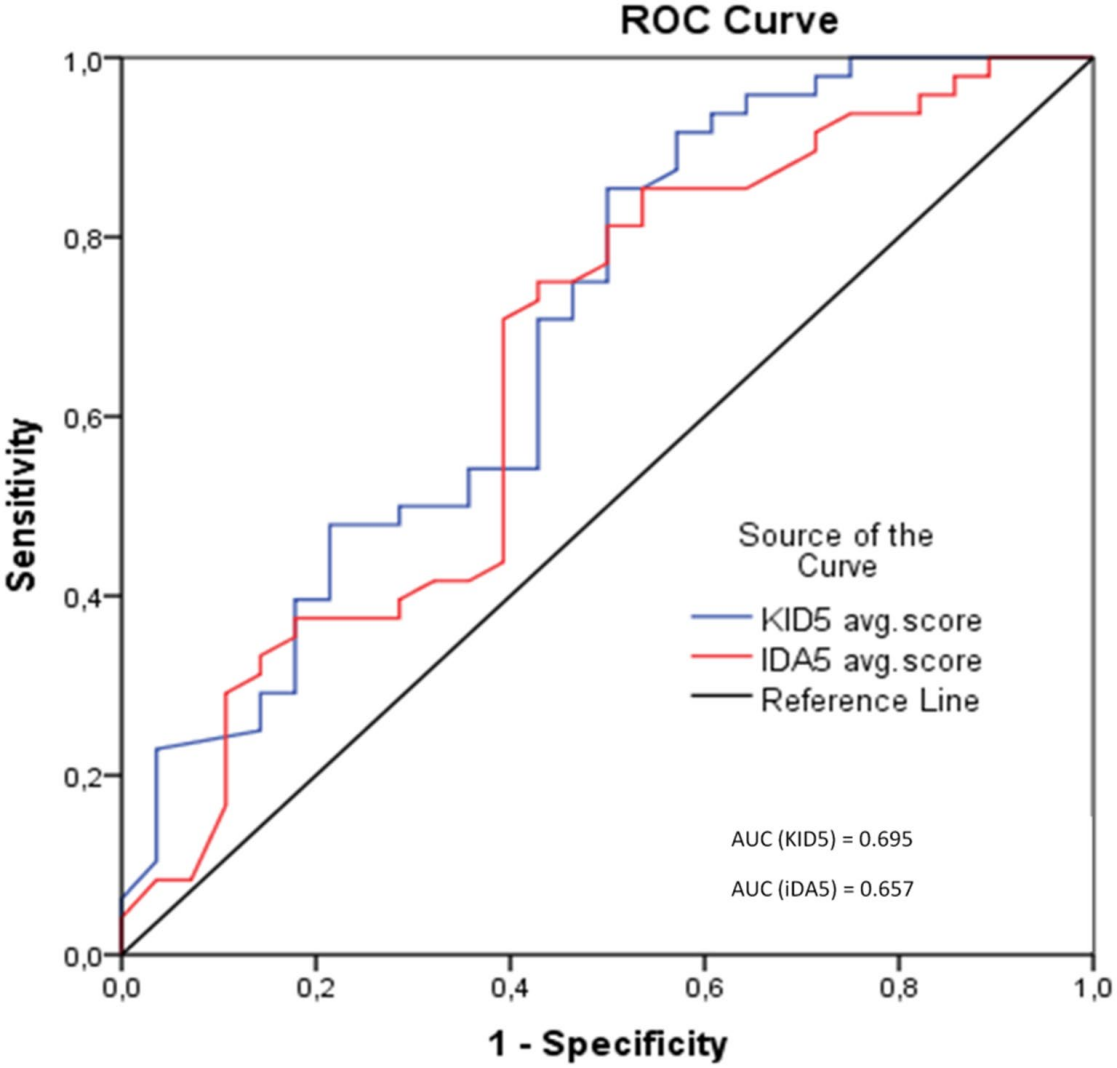
Receiver operating characteristic (ROC) curves for KID5 and iDA5 score, respectively. The AUC values are depicted. Diagonal segments are produced by ties

ROC analysis revealed AUC values of 0.695 (p-value = 0.005) and 0.657 (p-value = 0.023) for KID5 and IDA5 score, respectively. For KID5 score, a cut-off point of 7.4 with 71% sensitivity and 57% specificity is determined. Accordingly, for IDA5 score, a cut-off point of 8.3 with 71% sensitivity and 61% specificity is calculated (Table 4). The respective ROC curves are presented in Fig. 4. Moreover, considering the calculated cut-off points, KID5 score shows a more conservative performance compared to iDA5 score given their similar predictive capability. Such observation suggests that a live birth can be efficiently predicted by either a relatively lower KID5 score or a relatively higher iDA5 score.

**Table 4:** ROC analysis presenting AUC values and the respective true positive (sensitivity) and true negative (specificity) rates for KID5 and iDA5 scores. The optimal cut-off point is achieved where the sensitivity and specificity values are close enough to the AUC value

	AUC	p-value	95% CI	cut off	True Positive	True Negative
KID5 score	0.695	0.005	[0.568–0.822]	7.4	0.71	0.57
IDA5 score	0.657	0.023	[0.524–0.789]	8.3	0.71	0.61

## Discussion

The improvement of the overall IVF success rates and especially the outmost clinical outcome which is live birth remains the most demanding challenge in assisted reproduction. Until recently, embryo selection was based solely on morphological assessment and developmental patterns (e.g., expansion degree). Embryo selection using time-lapse monitoring systems along with the development of predictive algorithms is a promising approach in assisted reproduction technologies, thus, allowing embryologists to utilize an objective tool that provides them with additional biological information to select the optimal embryos for transfer to improve implantation, pregnancy and live birth rates [8, 14, 27, 28].

This study focused on the evaluation of the differences between the manual morphokinetic annotation system (KIDScore) and the automated AI model (iDAScore) score predictions as grading methods of the blastocyst stage embryos. The current results denote that KID5 and iDA5 scores correlate well, revealing that there is a good efficiency of the AI in recognizing division and morphological patterns as compared to the experienced embryologist. Moreover, high KID5 and iDA5 scores are associated with the probability of a live birth following ET; however, a high KID5 score yields is associated with a higher average probability for a live birth compared to a respectively high iDA5 score. Interestingly, our results demonstrated that although KID5 and iDA5 scores are co-modified, KID5 yields more conservative scores when adjusted for maternal age and number of blastocysts compared to the iDA5 score, indicating that a given live birth can be safely predicted provided by either a relatively lower KID5 score or a relatively higher iDA5 score. KID5 score conservative performance has been also justified via ROC analysis.

Our results are in line with the findings of two recent studies that demonstrated KID5 score predictive properties for blastocyst stage embryos and higher ongoing pregnancy and live birth rates for KID5 score-selected embryos [24, 29]. Moreover, KIDScore predictive model was found to be significantly associated with the chance of live birth in single embryo transfer and an acceptable agreement between the model and conventional embryological evaluations [30]. Interestingly, KIDScore model exhibited a satisfactory performance in the prediction of pregnancy and live birth outcomes in advanced age patients, although KID5 scores were lower compared to those corresponding to younger patients [31]. KIDscore high predictive value with regard to live birth rates following IVF treatment was also proved in a recent retrospective study, thereby enhancing morphological embryos assessment with morphokinetic information [32]. In a retrospective single-center study, KIDScore functioned as a live birth predictor for blastocyst-stage embryos. Therein, KIDScore on day 5 was proposed for embryo selection with the highest ability to result in a live birth among the blastocysts characterized as clinically usable by the laboratory standard operating procedure [33]. Further evidence of the clinical efficiency of automated embryo scoring in achieving higher live birth rates has been recently disclosed. The KIDScore D5™ algorithm was shown to correlate with higher live birth rates compared to conventional morphology assessment; thus, it was proposed to function as a valuable, supportive prediction tool with the final decision being made by the assisted reproduction expert [34].

On the other hand, certain studies support that artificial intelligence algorithms have superior predictive potential over the manually annotated scoring models. In detail, iDAScore model has recently been shown to have an equal or better performance than the manually annotated KIDScore model. Berntsen and his coworkers justified such finding in that the iDAScore model was trained not only on the embryos selected for transfer, but also on the embryos that are unsuitable for either transfer or cryopreservation [23]. Retrospective studies elaborated on the distribution of artificial intelligence model in the optimization of selecting the most viable embryo for transfer in terms of fetal heartbeat pregnancy which is a proxy for live birth [14]; especially in young patients, iDAScore was proposed as an optimal prediction model after single vitrified blastocyst transfer [35]. A recent multi-centre retrospective cohort study showed that iDAScore significantly surpassed the performance of KIDScore on day 5 embryos, with AUC determination proving that outperformance (AUC (KIDScore D5) = 0.645 and AUC (iDAScore v1) = 0.672) [36].

As thoroughly discussed, AI models and deep learning-related methods used for optimum embryo selection are often accompanied by potential biases. In detail, the AI models training on unbalanced data, the lack of generalizability across clinics due to single clinic-studies along with the limited performance metrics reported, may impair the clinical applicability of AI-based algorithms; thereby, AI model assessments on different datasets (i.e., incubation time, developmental stage and quality) are difficult to compare [37, 38].

The present study bears specific limitations including those of its retrospective design. Importantly, the high pregnancy rate could be considered biased due to optimal embryos selection based on the highest KID5/iDA5 scores for ET along with the strict exclusion criteria implementation that formed a good prognosis cohort. The small sample size of the present study is another reasonable limitation, since more data are needed to enhance the robustness of the presented results. Different approaches in terms of the number of transferred blastocysts per ET were co-analyzed herein and although this heterogeneity in the number of embryos does not provide a direct insight of the embryo scoring efficiency, it represents real world laboratory practice for all countries that legally allow the transfer of more than one embryo in a single FR/TH cycle thus reflects the efficiency of this prediction system in actual practice. A planned future study with the incorporation of a large set of cycles will incorporate the subgroup analysis of the cycles according to the number of embryos transferred per ET. Larger randomized controlled trials will offer an appropriate and valid evaluation of AI model performance.

Overall, either KIDScore or iDAScore are very useful supportive tools in successful live birth prediction where single embryo transfers occur. The current approach in assisted reproductive technology is that artificial intelligence predictive tools should be used in conjunction with the conventional embryological assessment and incorporated into the assisted reproduction routine application in order to support embryo selection and enhance the potential of IVF success improvement. Undoubtedly, AI-based embryo selection model eliminates biases sourcing from inter-laboratory and intra-laboratory variability; however, it should be further evaluated for its reliability, reproducibility and clinical actionability [39]. Until then, the contribution of well trained and experienced embryologists remains absolutely necessary at all steps of assisted reproduction.

## Data Availability

The dataset supporting the conclusions of this article is included within the article and the additional files.
